# Usutu Virus Africa 3 Lineage, Luxembourg, 2020

**DOI:** 10.3201/eid2805.212012

**Published:** 2022-05

**Authors:** Chantal J. Snoeck, Aurélie Sausy, Serge Losch, Félix Wildschutz, Manon Bourg, Judith M. Hübschen

**Affiliations:** Luxembourg Institute of Health, Esch-sur-Alzette, Luxembourg (C.J. Snoeck, A. Sausy, J.M. Hübschen);; Administration des Services Vétérinaires de l’Etat, Ministère de l’Agriculture, de la Viticulture et du Développement rural, Luxembourg (S. Losch, F. Wildschutz, M. Bourg)

**Keywords:** Usutu virus, blackbird, Luxembourg, phylogeny, viruses, zoonoses

## Abstract

We detected Usutu virus in a dead Eurasian blackbird (*Turdus merula*) in Luxembourg in September 2020. The strain clustered within the Africa 3.1 lineage identified in Western Europe since 2016. Our results suggest maintenance of the virus in Europe despite little reporting during 2019–2020, rather than a new introduction.

West Nile virus (WNV) and Usutu virus (USUV), members of the family *Flaviviridae*, share several epidemiologic traits and cocirculate in Europe. Both viruses are maintained through a transmission cycle involving bird and mosquito vectors. Migratory birds likely play a role in long-distance spread of USUV, similarly to WNV, and in the recent introduction of the virus to Europe from Africa (*1*).

In Europe, USUV has been associated with bird dieoff events since 2001 (*2*) and seems notably pathogenic for passerines and owls (*3*). Massive dieoff events of Eurasian blackbirds (*Turdus merula*) have become a hallmark of USUV circulation in Western Europe, enabling its detection through passive surveillance (*2*,*4*,*5*).

WNV and USUV are also occasionally transmitted through a mosquito bite to mammals (such as humans or horses), which are considered dead-end hosts (*3*) and experience a wide range of clinical signs up to neuroinvasive syndromes. Although most persons infected with USUV experience no or limited symptoms, USUV can cause more severe disease in certain persons or be detected in blood donations with yet-unknown consequences for the blood product recipients (*6*). The apparent intense virus circulation in countries neighboring Luxembourg that began in 2016, coupled with accumulating reports of USUV infections in humans (*7*), prompted us to initiate passive surveillance in Luxembourg as an early warning system for mosquitoborne *Flaviviridae* circulation.

During October 2018–September 2020, a total of 61 samples from 33 birds (Table) were submitted for investigation of WNV or USUV infection. The animals were found dead or died shortly after arrival at a wildlife rehabilitation center. All samples were screened for the presence of WNV and USUV by real-time reverse transcription PCR (Appendix). All tested negative for WNV. In September 2020, one brain sample from a Eurasian blackbird found dead in a home garden near the capital city tested positive for USUV (cycle threshold 22.09) (Table). Before death, the animal exhibited neurologic symptoms (disorientation, loss of coordination). The presence of USUV RNA was confirmed by a second real-time reverse transcription PCR test, and the whole genome was sequenced for further strain characterization.

**Table:** Samples collected in the framework of WNV and USUV passive surveillance, Luxembourg, 2018–2020*

Year	Bird species	Location	No. samples tested	Sample types	No. birds positive/no. total	
					WNV	USUV
2018	*Turdus merula*	Rehabilitation center	4	Liver, brain, kidney, heart	0/1	0/1
	*Tyto alba*	Rehabilitation center	6	Liver, brain, kidney, heart, tracheal swab, cloacal swab	0/1	0/1
	*Pica pica*	Esch-sur-Alzette	4	Liver, brain, kidney, heart	0/1	0/1
2019	*T. merula*	Rehabilitation center	10	Brain	0/10	0/10
	*Corvus corone*	Rehabilitation center	2	Brain	0/2	0/2
	*Corvus frugilegus*	Rehabilitation center	3	Brain	0/3	0/3
	*Corvus* sp.	Rehabilitation center	1	Brain	0/1	0/1
2020	*Sturnus vulgaris*	Lamadelaine, Pétange	20	Brain, tracheal swab, cloacal swab	0/9	0/9
	*Corvus* sp.	Pétange	10	Brain, tracheal swab, cloacal swab	0/4	0/4
	*T. merula*	Strassen	1	Brain	0/1	1/1
Total			61		0/33	1/33

*USUV, Usutu virus; WNV, West Nile virus.

Phylogenetic analyses assigned the USUV strain from Luxembourg to the Africa 3 lineage. This lineage was first identified in Germany in 2014 (*4*); since then, it has been regularly described in Belgium, France, Germany, and the Netherlands (*4*,*5*) and has occasionally been reported in the Czech Republic (2018) (*8*) and the United Kingdom (2020) (*9*) (Figure). More precisely, the USUV strain from Luxembourg grouped within the Africa 3.1 sublineage, which is the least represented lineage (*5*). It clustered together with strains from blackbirds and a common scoter (*Melanitta nigra*) detected in Belgium, Germany, France, and the Netherlands in 2016 and 2018 (Appendix Figure). The intermingling of the only 2 strains reported in 2020 from Luxembourg and the United Kingdom within Africa 3.1 and 3.2 together with earlier Western Europe strains suggests local virus spread rather than a new virus introduction in Europe. However, little reporting in 2019 and 2020 and the lack of sequences from Africa hamper definite conclusion. The time gaps between the estimated ancestors of the Africa 3 lineage (2009) and Europe 3 lineage (2002) (*5*) and the earliest sequences available (2014 for Africa 3 and 2010 for Europe 3) further suggest that silent USUV circulation is not uncommon. In addition, passive surveillance in Luxembourg might have missed earlier cases, as was reported in Austria, where only an estimated 0.2% of blackbirds killed by USUV were identified during 2003–2005 (*10*).

**Figure:**
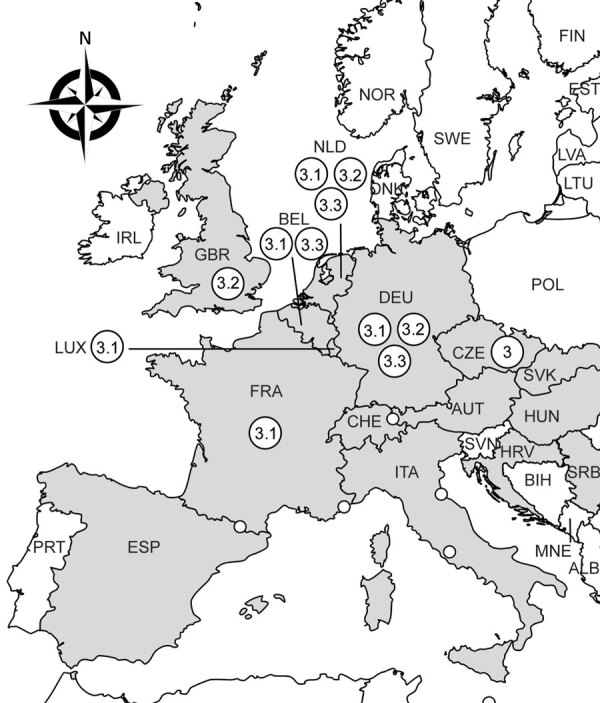
Geographic distribution of Usutu virus Africa 3 lineage in Europe. Countries are identified by 3-letter International Organization for Standardization codes (https://www.iso.org); gray indicates those where Usutu sequences were reported (partial E gene, partial NS5 gene, or complete polyprotein coding viral sequences available on GenBank). Large white circles indicate locations where Africa 3 lineage has been identified; sublineages are indicated within circles. Only partial NS5 sequence was available for Africa 3 strain from Czech Republic, preventing sub-lineage attribution. Small white circles delineate European microstates (AND, MCO, LIE, SMR and Vatican city); no Usutu virus circulation was reported. Map created with https://www.mapchart.net.

The transmission of both WNV and USUV is governed by a combination of factors, such as temperature, which influences both the developmental cycles of mosquitoes and virus transmissibility (*10*). Unusually high temperatures likely promoted the unprecedented USUV circulation in Western Europe (*4*,*10*). Expanding USUV geographic distribution is considered by some to be an indicator of WNV dispersion potential (*11*,*12*). The spread of WNV to Germany in 2018 and the Netherlands in 2020 corroborates this hypothesis. Because of the increasing frequencies of climatic anomalies, Luxembourg is also at risk for WNV to be introduced. Surveillance of mosquitoborne viruses such as USUV and WNV in animal hosts should be maintained and strengthened in the country as an early warning system to inform public health authorities.

Appendix Additional information about Usutu virus Africa 3 lineage, Luxembourg, 2020
