# Supplementary material for: Usutu Virus Africa 3 Lineage, Luxembourg, 2020
Source: Emerg Infect Dis. 2022 May;28(5):1076–9. doi: 10.3201/eid2805.212012 (PMC9045450; doi:10.3201/eid2805.212012)
Supplement: Appendix — Additional information about Usutu virus Africa 3 lineage, Luxembourg, 2020 [file 21-2012-Techapp-s1.pdf]

Article DOI: https://doi.org/10.3201/eid2805.212012

Usutu Virus Africa 3 Lineage, Luxembourg, 2020

Appendix

Material and Methods

WNV and USUV Detection

Upon sample reception, nucleic acids were extracted from 140 µL of virus transport medium in which cloacal and tracheal swabs were stored immediately after sample collection using the QIAmp viral RNA mini kit (Qiagen, Venlo, the Netherlands). Tissues (30 mg; brain, liver, kidney, heart) (Table, https://wwwnc.cdc.gov/EID/article/28/5/21-2012-T1.htm) were lysed in 600 µL of RLT lysis buffer supplemented with 14.3 M β-mercaptoethanol in the TissueLyser II (Qiagen) with 5 mm steal beads for 2x 30 sec at 30 Hz. After centrifugation for 3 min at 16 000 g to remove tissue debris, nucleic acids were purified from the supernatant using RNeasy mini kit (Qiagen) according to the manufacturer's instructions.

All samples were then tested for the presence of WNV RNA by a real-time reverse-transcription PCR suitable for lineage 1 and 2 detection and targeting NS2A (*1*). The assay was carried out with QuantiTect Probe RT-PCR kit (Qiagen) in a total reaction volume of 25 µL with 5 µL of RNA, 0.8 µM and 0.25 µM final concentrations of primers and probe respectively. Cycling conditions were: 50°C for 30 min, 95°C for 15 min followed by 45 cycles at 95°C for 30 sec, 55°C for 30 sec and 72° for 30 sec. USUV RNA was detected by a rRT-PCR targeting NS5 (*2*). The assay was carried out with QuantiTect Probe RT-PCR kit (Qiagen) in a total reaction volume of 25 µL with 5 µL of RNA, 0.8 µM and 0.2 µM final concentrations of primers and probe respectively, and supplemented with a final concentration of 1 mM $MgCl_2$. Cycling conditions were 50°C for 30 min, 95°C for 15 min followed by 45 cycles at 95°C for 15 sec, 60°C for 30 sec. USUV positivity was confirmed by a second rRT-PCR assay targeting NS1 (*3*). The PCR reaction and cycling conditions were similar to the initial USUV screening assay, except for 0.4 µM final concentration of primers and no additional $MgCl_2$.

USUV Genome Sequencing

RNA from the positive brain sample was first reverse transcribed into cDNA using Superscript III Reverse transcription (Invitrogen, Merelbeke, Belgium) and random hexamers (Invitrogen). The complete polyprotein coding sequence of USUV was then sequenced by amplifying overlapping amplicons using previously published (*4*) or newly designed primers (Appendix Table). Purified amplicons were sequenced on an ABI 3130 Avant capillary sequencer (Applied Biosystems) using PCR primers as sequencing primers. Contigs were assembled with Geneious Prime v2019.1.1 (Biomatters, Auckland, New Zealand) by aligning individual sequences to a similar reference strain (GenBank KY294723) identified by blasting amplicon sequences covering NS5. The consensus sequence of the complete polyprotein coding region was then used for phylogenetic analyses (GenBank accession no. OU674388).

Phylogenetic Analyses

All USUV sequences publicly available on 03.08.2021 were downloaded from GenBank into Geneious software. The curated sequence set (after removing sequences not belonging to USUV and clones) contained 843 partial and complete polyprotein coding sequences. All strains were renamed as follows: GenBank accession number_host/country/isolate/year. Given the non-uniform sequencing coverage obtained in various studies, phylogenetic analyses were performed on all complete polyprotein coding sequences (10,305 bp; n = 296) as well as partial sequences to obtain the best representation of the phylogenetic relationship of the strain from Luxembourg. For this purpose, all sequences longer than 500 bp were aligned using MAFFT as implemented in Geneious software. The most commonly sequenced regions were included and comprised of partial NS5 (nucleotides 9088–9597, 510 bp; n = 376) and partial envelope sequences (nucleotides 1003–2067,1065 bp; n = 377). All sequences with unresolved regions (Ns) were removed from the alignments. Identical partial NS5 and envelope sequences were summarized by including only one unique sequence identified thanks to the DNACollapser tool available in FaBox (*5*). The best substitution model fitting each alignment was identified using MEGA v6.06 (*6*). The model with the lowest Bayesian Information Criterion, considered to best describe the substitution pattern observed in the alignment, was then implemented to calculate phylogenetic trees with the Maximum Likelihood method and 500 bootstrap replicates in MEGA v6.06. Preliminary phylogenetic analyses showed that trees based on partial sequences lacked the power to define some lineages and the Africa 3 sublineages with confidence (bootstrap values at main

lineage defining nodes <75% and polyphyletic lineages) and led to some classification discrepancies (data not shown). Therefore further analyses focused on phylogenetic trees calculated on complete polyprotein coding sequences (Appendix Figure).

References

1. Eiden M, Vina-Rodriguez A, Hoffmann B, Ziegler U, Groschup MH. Two new real-time quantitative reverse transcription polymerase chain reaction assays with unique target sites for the specific and sensitive detection of lineages 1 and 2 West Nile virus strains. J Vet Diagn Invest. 2010;22:748–53. PubMed https://doi.org/10.1177/104063871002200515

2. Weissenböck H, Bakonyi T, Rossi G, Mani P, Nowotny N. Usutu virus, Italy, 1996. Emerg Infect Dis. 2013;19:274–7. PubMed https://doi.org/10.3201/eid1902.121191

3. Jöst H, Bialonski A, Maus D, Sambri V, Eiden M, Groschup MH, et al. Isolation of Usutu virus in Germany. Am J Trop Med Hyg. 2011;85:551–3. PubMed https://doi.org/10.4269/ajtmh.2011.11-0248

4. Bakonyi T, Gould EA, Kolodziejek J, Weissenböck H, Nowotny N. Complete genome analysis and molecular characterization of Usutu virus that emerged in Austria in 2001: comparison with the South African strain SAAR-1776 and other flaviviruses. Virology. 2004;328:301–10. PubMed

5. Villesen P. FaBox: an online toolbox for fasta sequences. Mol Ecol Notes. 2007;7:965–8. https://doi.org/10.1111/j.1471-8286.2007.01821.x

6. Tamura K, Stecher G, Peterson D, Filipski A, Kumar S. MEGA6: Molecular Evolutionary Genetics Analysis version 6.0. Mol Biol Evol. 2013;30:2725–9. PubMed https://doi.org/10.1093/molbev/mst197

Appendix Table. Additional primers designed to sequence USUV complete polyprotein coding region

Primer name	Primer orientation	Primer sequence (5′-3′)
Usu1464F	Forward	CACAACTAGGAGCATCAC
Usu1805R	Reverse	TGCCCGAGAAAGACACTGGA
Usu1726R	Reverse	TGCTTTGTGGCATGGGGCT
Usu3489F	Forward	GGAATGGAGATAAGACCCATGA
Usu3880R	Reverse	CATCTGAAAGAATGCTGCCC
Usu3559F	Forward	AGTGACATGATTGATCC
Usu3854R	Reverse	AGCAGGATGTTCTCTTGG
Usu79F	Forward	AACACAGTGCCGGCAGTTT
Usu162F	Forward	ATGCTGAAACGCGGCATACC
Usu245R	Reverse	GGCCAGCACGAATCGCACT
Usu341F	Forward	CACGGCAATGAAACACCTG
Usu391R	Reverse	CCGATTGTTGACCACGTTGA
Usu467F	Forward	CATGACGGCTGTTTCA
Usu533R	Reverse	CATGTCAGTCGCGTTGATG
Usu673R	Reverse	CAATGTCTTCTGGGTCAT

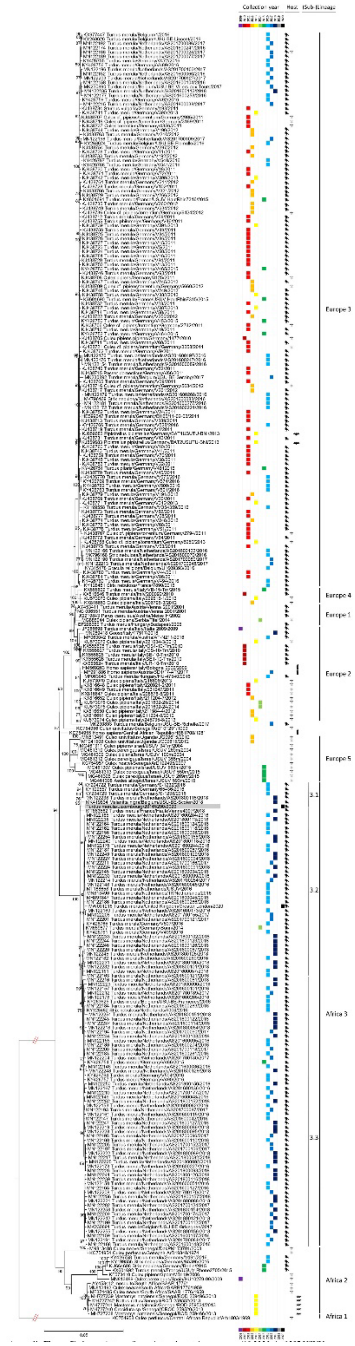

Appendix Figure. Phylogenetic tree of complete polyprotein coding sequences (10 305 bp) of 297 USUV sequences. The tree was calculated with Maximum Likelihood method and the GTR+G+I substitution model with 500 bootstrap replicates. Only bootstrap values ≥75% are shown. The scale represents the number of substitutions per site. For graphical representation, the long branch to the root of the tree was collapsed. The collection year(s) of the strains are represented with a color code spanning from 2009 to 2020 while hosts (bird, mosquito, human, bat, rodent) are shown with pictograms. The USUV strain from Luxembourg is highlighted with a gray box. Pictograms were designed by Freepik and used under license free terms.
